# Mammogram uptake and barriers among Palestinian women attending primary health care in North Palestine

**DOI:** 10.1080/13814788.2021.1985996

**Published:** 2021-10-11

**Authors:** Suha Hamshari, Zaher Nazzal, Mariam Altell, Israa Nanaa, Rawan Jbara, Ruba Sabri

**Affiliations:** a Family and Community Medicine Department, An-Najah National University, Nablus, Palestine; b Department of Public Health, Faculty of Medicine and Health Sciences, Nablus, Palestine; c Faculty of Medicine and Health Sciences, An-Najah National University, Nablus, Palestine

**Keywords:** Screening uptake, mammography, breast screening, barriers, breast cancer

## Abstract

**Background:**

Breast cancer affects women's lives worldwide, yet early detection is an effective strategy for reducing mortality. The participation of women in mammography screening is linked to their knowledge, attitudes and perceived barriers.

**Objectives:**

Our study aims to assess mammography screening uptake and barriers among women attending primary healthcare centres (PHCs) in northern Palestine.

**Methods:**

Using an interviewer administered questionnaire, we used a cross-sectional study design to determine mammography screening uptake, knowledge and barriers among 357 women attending PHCs in Northern Palestine between December 2018 and March 2019.

**Results:**

The mean age was 50 years. The majority (69.2%) were considered to have adequate knowledge about breast cancer and mammography screening. Mammography screening uptake among the participants was 37%. Almost 85% of the women had a positive attitude towards breastfeeding as a prophylaxis factor against breast cancer, while the most frequent barrier to mammography screening was that the participants believed they did not have any symptoms (28.6%), followed by 22.1% of them who did not want to know if they had breast cancer.

**Conclusion:**

The findings of this study highlighted the low mammography uptake among Palestinian women despite the adequate knowledge of those women and the fully accessible and free screening programme. Hence, interventional strategies should be implemented at several levels to enhance mammogram uptake.

KEY MESSAGES

- Breast cancer is the most prevalent cancer among women in Palestine. Despite a strong knowledge of mammography, it still has a poor uptake among women in primary health care centres.
- We need to find an efficient advisory policy to improve mammogram uptake among Palestinian women.

## Introduction

Breast cancer is the most commonly diagnosed cancer and the second leading cause of cancer-related deaths among women. It accounts for 11.7% of all cancer cases, with an estimated 2.3 million new cases in 2020 [1]. It strikes women at any age, but its incidence increases at the age of 40 [2]. In Palestine, according to the 2018 Annual Health Report, the incidence of breast cancer among females is 40 per 100,000, accounting for 27.6% of all cancer diagnoses among females [3].

People's willingness to engage in health-promoting practices is influenced by their health beliefs. The Health Belief Model (HBM), a social-psychological model, is a widely used conceptual framework for breast cancer screening [4]. It is utilised for explaining and predicting preventative behaviours such as screening. Countries that have reduced breast cancer mortality credit the decline to more effective therapies combined with effective population screening programs to detect the disease. Early detection strategies improve cancer outcomes by providing care at the earliest feasible stage, allowing for more successful, cost-effective and straightforward treatment [5]. Mammograms have proven to be an effective method of screening [6]. Marmot et al. [6] found in a cohort study that women invited to the screening had a 20%-reduction rate in breast cancer mortality after a 13-year follow-up. Even though women who attended screening had a significant decrease in mortality, it is hard to conclude that this is because women who did not participate had a different background risk.

In 2018, the American Cancer Society (ACS) recommended that women with an average risk of breast cancer aged 45–55 undergo annual screening mammography, and every other year for women aged 55 and above, while women aged 40–44 should have the opportunity to begin annual screening. However, the Palestinian guideline is to begin screening at the age of 40 every other year and then every year over 50 [2]. Furthermore, the Palestinian Ministry of Health (MOH) offers mammogram screening free of charge to all women aged 40 and above as well as younger women at high risk of breast cancer.

This study aims to assess the mammogram screening uptake among Palestinian women aged 40 and above who visit primary healthcare centres (PHCs) in the West Bank. In addition, we try to assess women's knowledge and attitudes towards mammogram screening and explore the barriers that prevent women from getting mammograms.

## Materials and methods

### Study design and setting

This study conducted a cross-sectional analysis of mammogram screening uptake collected from 357 women aged 40 and above attending Palestinian PHCs from December 2018 to March 2019.

### Recruitment of primary healthcare centres

PHCs were located in the West Bank's northern governorates, namely those of Nablus, Jenin, Tulkarem, Tubas, Qalqiliya and Salfit centres under the administration of the Palestinian MOH. Those were chosen first because they are usually the first contact point between most women in those communities and healthcare services. Second because they provide mammogram screenings free of charge.

The study population comprised all women over the age of 40. The minimum sample size required for the study was calculated to be 350 women at a 95% confidence level, with an effect size of 20% and a 0.05 absolute precision on either side of the proportion (p). Four hundred women were approached and invited to participate in the study; 377 agreed to participate, with 20 leaving before completing their questionnaires, resulting in a sample size of 357.

### Recruitment of participants

The study's participants were chosen using a convenient sampling technique based on proportional allocation. We chose females in proportion to the average number of female attendants in each PHC each month at each directorate, covering the whole working day. We included in the study all females attending PHCs for preventative or therapeutic services or accompanying a client. Those with a previous history of breast cancer or breast surgery were excluded.

### Questionnaire development and data collection

An interviewer administered questionnaire was used to collect data. It was built on an extensive review of similar studies [7–11], as well as a previous 2016 study in the West Bank that targeted Palestinian female HCWs [12].

The questionnaire collected different categories of data and consisted of four parts. First, it asked about the women's demographic characteristics, including age, education, marital status and the family's history. The second part investigated their knowledge of mammograms; 19 questions were included, with one score for each correct response and zero for incorrect responses. We computed the knowledge score for each participant by adding the right answers, dividing them by the total number of knowledge questions, and multiplying the result by 100%. We reported the results as poor (< 50%), fair (50%–70%) and excellent (> 70%). The third part sought responses from participants regarding their attitudes concerning mammogram screening (four questions). This part was prepared based on the constructs of the HBM [13]. It sets up frameworks for perceived risk, benefit, self-efficacy and barriers [6,14]. The last part collected data on the barriers affecting mammography screening uptake such as fear, embarrassment, being busy, not having symptoms, mammograms side effects, etc.

Three experts in the field reviewed the questionnaire, and we conducted a pilot study on 20 women attending PHCs to evaluate the questionnaire's clarity, understanding and feasibility. The data collected during the pilot were used to improve the quality and efficiency of the main survey. Those who participated in the pilot study were excluded from the larger sample.

### Statistical analysis

Statistics Package for Social Sciences (SPSS) was used for data entry and analysis. Descriptive statistics of the responses were generated. A Chi-square test was used to assess the statistical significance of group categories, and p values of < 0.05 were considered statistically significant.

### Research ethics

Ethical approval to conduct the study was obtained from the Institutional Review Board (IRB) of An-Najah National University, as well as permission from the Palestinian MOH. Participants' privacy and confidentiality were assured. A written consent and study objectives were attached to each questionnaire, and the participants were informed that participation in the study was voluntary.

## Results

### Background characteristics

A total of 357 participants completed and returned the questionnaire giving an overall response rate of 89.3%. The demographic profile and general information are included in Table 1. The average age was 50 years, with a standard deviation of 8 years. More than half (56%) had obtained a higher education level, while the remainder had a high school diploma or lower. For married women, the majority of their husbands were employed (77%), and the income level was divided into low income (30%), moderate (52%) and high income (18%).

**Table 1.** Demographic characteristics of the participants (*N* = 357).

Characteristics	(Mean ± SD)
**Age**	50.77 ± 8.43
**Age at first delivery**	20.89 ± 4.34
	**Frequency (%)**
***Profession***	
Housewife	277 (77.6%)
Working	80 (22.4%)
***Academic degree***	
Diploma or less	158 (44.3%)
Higher education	199 (55.7%)
***Marital status***	
Married	267 (74.8%)
Single	40 (11.2%)
Divorced/ widow	50 (14%)
***Income***	
Low (<1.000)	108 (30.3%)
Moderate (1.000–3.500)	186 (52.1%)
High (> 3.500)	63 (17.6%)
***Husband's profession^a^***	
Working	200 (77%)
Not working	60 (23%)
***Husband's academic degree^a^***	
Diploma or less	251 (96.5%)
Higher education	9 (3.5%)
***Parity status***	
Yes	290 (94.2%)
No	18 (5.8%)
***Breast feeding***	
Yes	271 (92.2%)
No	23 (7.8%)
***Type of family***	
Nuclear family	273 (92.5%)
Extending family	22 (7.5%)

^a^These questions were not applicable to all participants.

Another set of questions tackled the maternal history of the respondents. Of the respondents, 94% had given birth; the average age at birth was 21 years old with a standard deviation of 4 years. The majority of the women had breastfed (92%) and lived in a nuclear family setting (93%).

### Knowledge and attitudes

The study showed that the participants had an adequate level of knowledge about breast cancer and mammogram screening. Most women (88%) said they knew that early breast cancer detection increases survival rates. They also had prior knowledge that breast cancer was the most common cancer among women (85%) and that it was recommended to perform a breast self-exam monthly (78%). On the other hand, less than half (44%) were aware that obesity increases the chances of breast cancer, and only a quarter of the respondents knew that the birth of a first child at an age over 30 increases the risk of breast cancer (25%).

Most women (73%) understood what mammogram screening was, and 75% were aware that mammography is one of the most important methods for early detection of breast cancer. More than half (55%) answered correctly about the recommended age to start having mammograms. However, only 32% of them responded correctly to the question regarding the recommended frequency. The frequency and percentages of all the knowledge questions are summarised in Table 2.

**Table 2.** Knowledge of breast cancer among the participants (*N* = 357).

*Knowledge statements*	Yes (%)	No (%)
Early detection of breast cancer increases survival rates	314 (88.0%)	43 (12%)
Breast cancer is the most common cancer among women	304 (85.2%)	53 (14.8%)
It is recommended to perform breast self-exam monthly	278 (77.9%)	79 (22.1%)
Breast cancer can occur in any age group	277 (77.6%)	80 (22.4%)
Late detection of breast cancer means the disease has metastasised to other parts of the body	270 (75.6%)	87 (24.4%)
Mammography is one of the most important methods for the early detection of breast cancer	269 (75.4%)	88 (24.6%)
Definition of mammography	262 (73.4%)	95 (26.6%)
The recommended age to start having mammograms	197 (55.2%)	160 (44.8%)
Smoking is a risk factor for breast cancer	244 (68.3%)	113 (31.7%)
The risk of breast cancer increases with age	232 (65%)	125 (35%)
Breast cancer may have a genetic predisposition	221 (61.9%)	136 (38.1%)
Breast cancer can develop without symptoms	210 (58.8%)	147 (41.2%)
Obesity increases the risk of breast cancer	157 (44.0%)	200 (56.0%)
Contraceptive pills increase the risk of breast cancer	143 (40.1%)	214 (59.9%)
Null parity is a risk factor for breast cancer	133 (37.3%)	224 (62.7%)
Menopause after age 50 increases the risk of breast cancer	94 (26.3%)	263 (73.7%)
Menarche before age 11 increases the risk of breast cancer	91 (25.5%)	266 (74.5%)
The birth of the first child at an age older than 30 increases the risk of breast cancer	89 (24.9%)	268 (75.1%)
The recommended frequency for mammography screening	115 (32.2%)	242 (67.8%)
Attitude statements	Agree	Disagree
Breastfeeding can protect against breast cancer	305 (85.4%)	52 (14.6%)
Mammography can detect breast cancer before its symptoms appear	196 (54.9%)	161 (45.1%)
Mammography has adverse effects	196 (54.9%)	161 (45.1%)
Mammography is painful	144 (40.3%)	213 (59.7%)

After the score of knowledge about breast cancer and mammography was computed for each participant, the results showed that most of the participants (46%) had fair knowledge, 23% had excellent knowledge, and 31% had a poor score of knowledge about breast cancer and mammography.

The questions examined the attitude towards mammograms in Table 2, 85.4% of the women agreed that breastfeeding protects against breast cancer, and 54.9% believed that mammograms help in the early detection of cancer. However, some thought mammograms have their side effects or that it is dangerous and the procedure is painful.

### Mammogram uptake

Results showed that the mammogram uptake was 37.0% (95%CI= 3.2%–4.2%) and that the highest justification for non-uptake is that women believed there is no need for the screening if they did not display any symptoms (29%). This was followed by them not wanting to know whether they had breast cancer or not (22%) and being busy (16%). Table 3 lists all the barriers ranked by frequency.

**Table 3.** Barriers preventing women from uptaking mammogram screening (*N* = 255).

Barrier	Frequency (%)
I do not have any symptoms	102 (28.6%)
I do not want to know whether I have breast cancer or not	79 (22.1%)
I am very busy	57 (16%)
I do not think that I might have breast cancer	56 (15.7%)
I am suffering from other chronic diseases that I am running for	36 (10.1%)
I am very shy to expose my breasts	29 (8.1%)
There are no guidelines or recommendations by the MoH	26 (7.3%)
Mammography is painful	24 (6.7%)
I do not know about it	19 (5.3%)
Mammography causes adverse effects	15 (4.2%)
Early detection is not important because cancer can't be treated	11 (3.1%)
I do not have trust in primary health care staff	8 (2.2%)
Mammograms can't detect breast cancer before its symptoms appear	6 (1.7%)

This study also studied the relationship between mammography uptake and participants' characteristics (Table 4). The results showed a significant difference in mammography uptake due to knowledge; participants with an excellent or fair level of knowledge have a higher rate of mammogram uptake (*p* = 0.038). Perceived importance of mammograms and perceived benefits of early detection were also found to significantly predict higher levels of mammogram uptake (*p* = 0.020 and < 0.001, respectively). The uptake was higher among married participants, nursing women and those who had not given birth. On the other hand, there was no significant difference in mammogram uptake screening when comparing participants’ profession, academic degree, income, parity status, breastfeeding and marital status (*p* < 0.05).

**Table 4.** Characteristics of participants in relation to mammography screening uptake (*N* = 357).

Characteristics	Uptake *N* = 132 (37%)	No uptake *N* = 225(63%)	*p* value*
**Profession**			
Housewife	107 (39%)	170 (61%)	0.229
Working	25 (31%)	55 (69%)	
**Academic degree**			
Diploma or lower	56 (35.4%)	102 (64.6%)	0.593
Higher education	76 (38%)	123 (62%)	
**Marital status**			
Married	100 (37.5%)	167 (62.5%)	
Single	13 (32.5%)	27 (67.5%)	0.822
Divorced /widow	19 (38%)	31 (62%)	
**Income**			
Low (< 1000)	35 (32%)	73 (68%)	
Moderate (1000–350)	78 (42%)	108 (58%)	0.123
High (> 3500)	19 (30%)	44 (70%)	
**Parity status**			
Yes	110 (38%)	180 (62%)	0.935
No	7 (39%)	11 (61%)	
**Breastfeeding**			
Yes	105 (39%)	166 (61%)	0.229
No	6 (26%)	17 (74%)	
**Knowledge**			
Poor	30 (27.3%)	80 (72.7%)	
Fair	67 (40.6%)	98 (59.4%)	0.038
Excellent	35 (42.7%)	47 (57.3%)	
**Perceived benefits of the monthly exam**			
Agree	104 (37.4%)	174 (62.6%)	0.749
Disagree	28 (35.4%)	51 (64.6%)	
**Perceived benefits of early detection**			
Yes	123 (39.2%)	191 (60.8%)	0.020
No	9 (20.9%)	34 (79.1%)	
**Perceived importance of mammograms**			
Yes	114 (42.5%)	155 (57.6%)	< 0.001
No	18 (20.5%)	70 (79.5%)	
**Perceived severity of the breast cancer**			
Yes	106 (39.3%)	164 (60.7%)	0.115
No	26 (29.9%)	61 (70.1%)	
**Perceived barriers** (it is painful)			
Yes	35 (46.1%)	41 (53.9%)	0.065
No	97 (34.5%)	184 (65.5%)	
**Perceived barriers** (it has side effects)			
Yes	9 (24.3%)	28 (75.7%)	0.092
No	123 (38.4%)	197 (61.6%)	

*Chi-squared test.

## Discussion

### Main findings

Our study was conducted on Palestinian females over the age of 40 to assess mammography screening uptake and to assess knowledge, attitude and barriers to mammography screening among women.

Although nearly two-thirds of the 357 women had robust knowledge, such knowledge did not translate into a comparable level of uptake. The absence of symptoms was the most common reason that women avoided getting mammograms.

### Comparison with existing literature

The rate of mammogram uptake among participants was 37%. This is higher than the results of similar studies conducted in Malaysia (31%) [7], Jordan (12.4%) [15] and Turkey (11%) but lower than the UAE (55.9%) or Iran (44.3%) [4,16,17]. A previous study conducted on health care workers (HCW) in Palestine shows that 50% of HCW perform mammography screening [12], which is higher than the mammography uptake among women attending PHCs. This may be due to the greater perceived benefits of mammography screening among HCW. This outcome is directly related to a delay in seeking help, followed by a late diagnosis, poor survival, and increased management costs, particularly in countries with a scarcity of breast cancer treatment centres.

Concerning mammogram screening, an interesting difference from the above was the high number of women who were aware of mammogram screening (73%) and its importance (75%), on the one hand, and the percentage of those who adequately responded to the recommended frequency (32%), on the other hand. However, one result which might explain that pertains to the fact that only 9.2% of the participants indicated that workshops were one of the sources of their knowledge about breast cancer and mammography screening.

Results indicated that most participants (69%) had adequate knowledge (either excellent or fair) about breast cancer and mammography screening. This result was higher than reported in Uganda (29%) [18], Yemen (50%) [8] and Lebanon (55%) but lower than that in Turkey (77%) [4,9].

Despite the latter being a positive outcome, the results also show a gap between the knowledge of screenings and the actual uptake. Although most participants (69%) had adequate knowledge about breast cancer and mammography screening, only 37% of them had taken a mammogram screening. This is another indicator that although knowledge is necessary to encourage women to take the screening, it is not sufficient by itself, as other studies have shown [10,17]. This finding confirms the necessity of an organised programme at the community and healthcare services levels. A systematic review found that a systematic screening programme utilising letters, phone reminders and scheduled appointments would significantly increase screening participation [11].

On the one hand, the negative barriers (attitudes) are illustrated in fear of the adverse side effects of mammograms and the anxiety of pain during the procedure. Appropriate education and training that promote health beliefs as well as information on breast cancer screening will alleviate fear, increase self-efficacy and encourage mammography use. On the other hand, there were also optimistic attitudes such as females’ perceived importance of mammograms and perceived benefits of early detection. Other researchers in Saudi Arabia have revealed similar results [13,14].

The investigation of barriers to mammography screening uptake in the current study was based on the constructs of the HBM. The results show that reasons preventing women from uptaking mammogram screening were that they believed they did not have any symptoms (28.6%), followed by 22.1% of them who did not want to know whether they had any symptoms of breast cancer or did not. In two examples from bordering Jordan, women did not take the screening because they believed they did not have any health problem (74.8%) or because of the fear of results (63.8%) [15,19]. These findings are consistent with cancer fatalistic beliefs, which claim that an external force controls all events or actions in a person's life [20]. An inverse relationship between cancer fatalistic attitudes and mammography uptake has been observed in the literature [21,22]. This finding emphasises the significance of further investigating Palestinian women's perceived cancer fatalistic attitudes and discussing them as part of cancer prevention programs. Campaigns so far have focussed on detecting breast cancer through symptoms rather than on its curable process. Furthermore, well-structured educational programmes to cross those barriers with consideration given to the social and cultural factors are mandatory among those Palestinian women.

### Limitations

Despite the high response rate, there are some limitations to this study that should be considered. First, the study's cross-sectional design limits the causal inferences about mammogram uptake. Second, the study only included PHC attendees, limiting the generalisability of the findings to this population subgroup. Women who attend PHCs may have a higher level of mammogram knowledge than the general population. On the other hand, PHCs are located throughout Palestine and provide a wide range of comprehensive preventative and curative health services at no cost, making them accessible to the vast majority. Furthermore, using a convenient sample approach to select participants may limit the generalisability of these results.

## Conclusion

Despite the strong knowledge of breast cancer and mammography, mammography uptake for early breast cancer detection remains low. High-level authorities are invited through initiatives such as community health promotions and worship programmes to decrease barriers to mammography by raising women's willingness to undergo mammography. A well-structured screening programme should be implemented at multiple levels, including individuals, communities and health care organisations. Educational interventions supported by individual letters, automated phone message reminders, and scheduled visits as well as effective interventions to remove barriers, particularly logistical ones, could all contribute to increased uptake.
